# Microscopic Pollen Image Classification via Contour-Signal Representation, Wavelet Analysis, and CNN

**DOI:** 10.3390/jimaging12070326

**Published:** 2026-07-18

**Authors:** Abror Shavkatovich Buriboev, Akhram Nishanov, Shuxrat Isroilov, Inomjon Narzullaev, Umidjon Djumayozov, Shavkat Buriboyev, Temur Azamov, Parda Yuldashov, Davron Shodmonov, Djamshid Sultanov, Abbos Abduvaytov

**Affiliations:** 1 Department of Exact Sciences, Kimyo International University in Tashkent, Tashkent 100121, Uzbekistan; 2 Department of Software of Information Systems, Tashkent University of Information Technologies named after Muhammad al-Khwarizmi, Tashkent 100084, Uzbekistan; 3 Department of Computing Systems Engineering, Samarkand State University, Samarkand 140104, Uzbekistan; 4 Department of Civil Engineering, Samarkand State Technical University, Samarkand 140143, Uzbekistan; 5 Agency Innovative Development, Tashkent 100174, Uzbekistan; 6 Department of Surgery, Samarkand State Medical University, Samarkand 140100, Uzbekistan; 7 Department of Computer Engineering, Samarkand Institute of Economics and Service, Samarkand 140100, Uzbekistan

**Keywords:** micro-object recognition, computer vision, wavelet transform, Fourier transform, convolutional neural network, image segmentation, hybrid models

## Abstract

Accurate classification of pollen grains in microscopic images remains challenging because of noise, structural variability, background complexity, weak texture, and intra-class similarity. To address these issues, this study proposes a hybrid framework that integrates contour-signal modeling, spectral–wavelet analysis, and deep learning for robust microscopic pollen image recognition. In the proposed approach, microscopic pollen images are first converted into contour-based point-signal representations, allowing object boundaries to be analyzed as structured one-dimensional signals. To improve signal quality under real imaging conditions, the framework incorporates Gaussian, median, and contour-aware filtering together with defect-point detection and correction. The processed contour signals are then analyzed using Fourier transform, continuous wavelet transform, and discrete wavelet transform to extract complementary global and local descriptors. These enriched representations are provided to a convolutional neural network for final classification. Experiments conducted on a seven-class microscopic pollen-image dataset demonstrate that the proposed method outperforms conventional computer-vision and baseline deep-learning approaches. The best-performing hybrid configuration achieved an error rate of 6.4%, while the overall classification accuracy reached 0.977 with an F1-score of 0.966, compared with 0.837 for a traditional computer-vision pipeline. These results confirm that combining contour-based signal processing with hierarchical deep feature learning provides an effective and noise-robust strategy for microscopic pollen image recognition. However, the present validation is limited to pollen images, and further experiments on broader microscopic object datasets are required to assess generalization to other micro-object categories such as nanoparticles, fibers, rods, and synthetic microstructures.

## 1. Introduction

Microscopic image analysis has become an essential component of modern scientific research and industrial inspection, supporting applications in palynology, microbiology, pathology, environmental monitoring, materials science, pharmaceutical manufacturing, and precision agriculture. Automated recognition of microscopic objects, including pollen grains, microorganisms, spores, cells, mineral particles, and synthetic microstructures, enables rapid quantitative analysis while reducing human subjectivity, labor-intensive manual inspection, and inter-observer variability. As advances in digital microscopy continue to improve image acquisition speed and resolution, there is an increasing demand for intelligent image-analysis systems capable of accurately recognizing microscopic objects under diverse and often challenging imaging conditions.

Although remarkable progress has been achieved in computer vision over the past decade, microscopic micro-object recognition remains fundamentally different from conventional object recognition [1,2]. Most modern object-recognition algorithms have been developed for natural-image datasets, where target objects occupy relatively large image regions, exhibit abundant texture, possess distinctive semantic information, and are surrounded by contextual cues that facilitate recognition. Deep convolutional neural networks have demonstrated outstanding performance under these conditions by learning hierarchical feature representations directly from image pixels [3,4,5,6,7,8]. However, these assumptions rarely hold for microscopic images. Microscopic objects usually occupy only a small portion of the image, contain weak or repetitive textures, exhibit low contrast, and often differ only in subtle morphological characteristics. Furthermore, discriminative information is concentrated primarily along object boundaries rather than throughout the entire object. Consequently, accurate recognition depends heavily on preserving contour morphology and fine structural details that can easily be degraded during image acquisition or segmentation.

Microscopic imaging also introduces challenges that are uncommon in ordinary object recognition. Sensor noise, optical blur, uneven illumination, staining variability, background artifacts, and limited depth of field frequently produce contour discontinuities, isolated defect points, irregular boundary deformation, and local intensity fluctuations. At the same time, microscopic datasets typically exhibit high intra-class variability caused by specimen orientation, deformation, and biological diversity, while inter-class differences are often extremely subtle. These characteristics significantly increase the difficulty of extracting robust discriminative features and limit the direct applicability of conventional object-recognition methods developed for natural images. Existing microscopic object-recognition methods generally rely on three complementary research directions: contour-based structural analysis, spectral signal representation, and deep-learning-based feature learning. Contour-based approaches effectively describe object morphology while reducing background interference, but their performance depends heavily on the quality of contour extraction and is easily degraded by segmentation errors or boundary defects. Spectral descriptors such as Fourier transform provide compact representations of global contour geometry; however, they cannot accurately characterize localized structural variations that frequently distinguish visually similar microscopic objects. Wavelet-based methods overcome this limitation by providing localized multi-scale analysis of contour signals, yet they remain handcrafted mathematical representations whose discriminative capability depends strongly on feature design and parameter selection. Deep convolutional neural networks automatically learn hierarchical feature representations and have significantly improved microscopic image classification; nevertheless, most existing CNN-based methods operate directly on raw microscopic images and do not explicitly compensate for contour degradation or exploit contour-specific structural information before feature learning. A detailed discussion of these representative approaches and their limitations is presented in Section 2.

The above observations indicate that no single methodology sufficiently addresses all challenges associated with microscopic micro-object recognition. Image filtering can effectively suppress sensor noise, contour distortion, and boundary discontinuities before feature extraction, thereby improving the stability of contour representation. Fourier transform provides compact global shape information but cannot preserve localized contour deformation. Wavelet transform complements Fourier analysis by simultaneously representing contour signals in spatial and frequency domains, enabling robust multi-scale characterization of local morphological variations. Finally, convolutional neural networks provide adaptive hierarchical feature learning and nonlinear classification that cannot be achieved by handcrafted descriptors alone. Rather than viewing these techniques as independent processing steps, they should be integrated into a unified framework in which each component compensates for the limitations of the others.

Motivated by these observations, this paper proposes a hybrid contour-signal-based framework for microscopic micro-object classification that systematically integrates contour quality enhancement, spectral–wavelet feature extraction, and deep representation learning. The proposed framework first transforms segmented object boundaries into normalized contour signals, applies adaptive filtering and defect-point correction to improve contour stability, extracts complementary global and local structural descriptors using Fourier and wavelet analysis, and finally performs hierarchical feature learning using a convolutional neural network. The novelty of this work does not lie in the individual application of filtering, Fourier transform, wavelet transform, or CNN, since each of these techniques has been extensively investigated in previous studies. Instead, the primary contribution is their systematic integration into a unified contour-signal analysis framework specifically designed to address the unique challenges of microscopic micro-object recognition, including weak boundaries, imaging noise, contour degradation, structural variability, and high intra-class similarity.

The principal contributions of this study are summarized as follows.

1. A normalized contour-signal representation for microscopic micro-objects. A contour-based point-signal modeling strategy is proposed to convert microscopic object boundaries into structured one-dimensional signals that preserve morphological information while reducing the influence of irrelevant background regions.
2. A hybrid contour-enhancement and spectral–wavelet feature extraction framework. Adaptive filtering, contour-defect correction, Fourier transform, and multi-scale wavelet analysis are integrated to generate complementary global and local structural descriptors that are robust to imaging noise and contour degradation.
3. A unified CNN-based microscopic micro-object classification framework. Enhanced contour-signal representations are combined with hierarchical deep feature learning to improve classification robustness and accuracy under challenging microscopic imaging conditions characterized by weak boundaries, structural variability, and high intra-class similarity.

The remainder of this paper is organized as follows. Section 2 reviews representative studies on microscopic object recognition, contour-based shape analysis, spectral signal processing, wavelet-based feature extraction, and deep-learning-based classification methods. Section 3 presents the proposed contour-signal representation, contour-enhancement strategy, spectral–wavelet feature extraction, and convolutional neural network architecture. Section 4 describes experimental datasets, implementation details, evaluation metrics, comparative experiments, and ablation studies. Finally, Section 5 concludes the paper and outlines directions for future research.

## 2. Related Works

Automated classification of microscopic micro-objects has become an important re-search direction in computer vision, biomedical image analysis, environmental monitoring, palynology, agriculture, and industrial inspection. Microscopic images usually contain small objects with complex boundaries, weak contrast, non-uniform illumination, background artifacts, noise, deformation, and high intra-class similarity. Therefore, reliable classification requires not only visual appearance analysis but also robust structural, spectral, and statistical representation of micro-object morphology. Basic image-processing principles, including filtering, segmentation, morphological processing, and feature extraction, have been widely discussed by Gonzalez and Woods [9]. Contour analysis and its application to image and signal processing were systematically studied by Tojiyev et al. [10], who showed that object boundaries can provide important structural information for recognition. In microscopic image analysis, Jumanov and Safarov proposed image-processing algorithms for micro-objects in medical diagnostic systems [11], while Gopal et al. [12] and Gandhi [13] demonstrated the importance of computer-vision methods in complex recognition environments. Although these studies confirm the usefulness of classical computer vision, their performance often depends strongly on preprocessing quality, illumination stability, and manually designed features.

Traditional computer-vision systems mainly rely on preprocessing, segmentation, contour extraction, handcrafted feature design, and statistical classification. Visual recognition systems have been applied in different practical domains, including terrain recognition [14], autonomous robotic vision [15], face detection and image analysis [16], and medical optical image processing [17]. These studies show that image-processing pipelines can extract useful geometric, morphometric, texture, and color descriptors. Groshev and Korolkov [18] discussed technical vision and image-processing systems, while Savelyeva and Smushkin [19] described forensic recognition methods based on visual features. In the context of micro-object recognition, Jumanov and Safarov optimized micro-object identification using a pyramidal model with image segmentation [20]. Matroushi [21] also studied object detection, recognition, and classification using computer vision and artificial intelligence approaches. These works provide important foundations for object recognition; however, classical pipelines often become less robust when objects have noisy boundaries, strong intra-class variability, or complex backgrounds.

Microscopic and industrial image-analysis systems require robust feature extraction under real imaging conditions. Zyuzin et al. [22] proposed a deep-learning-based computer-vision system for automatic asbestos control, showing the practical importance of visual recognition in microscopic and industrial inspection. Chen et al. [23] studied intelligent camera dust-removal control, highlighting that image quality and optical contamination can strongly affect recognition reliability. Russ [24] presented computer-assisted microscopy techniques for measuring and analyzing microscopic images, including shape, size, texture, and intensity features. More recently, Maier-Hein and Reinke [25] emphasized the importance of reliable validation methodology in image analysis, including careful metric selection, statistical validation, and appropriate experimental design. These studies indicate that microscopic image classification requires not only accurate models but also robust preprocessing, proper validation, and reliable performance evaluation. Contour-based and boundary-based methods are especially relevant for microscopic micro-object classification because many micro-objects, including pollen grains, cells, microorganisms, and mineral particles, are strongly characterized by external morphology. Wiskott et al. [26] demonstrated that structural graph-based representations can capture discriminative visual patterns for recognition. Novotorcev [27] studied algorithms for searching and restoring objects in aerial images, confirming that structural information can play an important role in recognition tasks. Vasilchenko [28] investigated mathematical and software support for computer-image processing in large databases, emphasizing the need for effective feature extraction and recognition algorithms. In microscopic images, boundary representations such as contour descriptors, chain codes, curvature features, Fourier descriptors, and radial-distance functions can reduce the influence of background pixels and focus attention on object shape. However, contour-based methods may be sensitive to segmentation errors, defective contour points, and local noise. Therefore, combining boundary representation with adaptive learning is a promising direction.

Fourier and wavelet transforms have been widely used to analyze signals, shapes, textures, and image structures. Hamad [29] investigated segmentation and recognition of medical images based on shearlet transforms and neural networks, showing the value of combining signal transforms with learning-based models. Bogush [30] studied combined block algorithms for detecting and tracking moving objects in video sequences, demonstrating the usefulness of combining multiple analytical components. Wang [31] developed software tools based on multiscale wavelet analysis for image processing and retrieval. Tang et al. [32] reviewed digital image-processing techniques for optical tweezers and emphasized the importance of signal-processing methods in microscopic imaging applications. In contour-based micro-object classification, Fourier transform can represent global frequency characteristics, while continuous and discrete wavelet transforms can capture local and multi-scale contour variations. Haar wavelets are useful for abrupt boundary transitions, while Daubechies wavelets are suitable for smoother structural patterns. Nevertheless, transform-based features alone are usually insufficient for complex classification tasks and should be combined with learning-based classifiers.

Segmentation and structural analysis provide another important foundation for micro-object recognition. Horowitz and Pavlidis [33] proposed image segmentation by tree traversal, while Haralick and Shapiro [34] presented classical image segmentation techniques that remain influential in computer vision. These studies established important principles for separating objects from background and extracting meaningful regions. In pollen and microscopic object recognition, Schiele et al. [35] applied neural networks for automated airborne pollen classification, demonstrating that learning-based approaches can improve recognition performance in palynology. Lin et al. [36] proposed feature pyramid networks for multi-scale object detection, and Girshick [37] introduced Fast R-CNN for object localization and recognition. These deep-learning-based works demonstrate the importance of hierarchical feature learning and multi-scale representation. However, in microscopic image classification, standard deep models may not always fully exploit contour-specific structural information, especially when training data are limited. Neural-network-based recognition methods have become dominant in many image-classification tasks. Druki [38] investigated neural-network detection and recognition of symbols on complex backgrounds, showing that neural networks can learn robust representations in difficult visual conditions. Kosaty [39] studied hybrid classifiers based on cascades and deep neural networks, indicating that combining different classification mechanisms can improve recognition reliability. Strotov [40] showed that combining multiple algorithms can increase detection and localization accuracy. Burns and Shulgan [41] discussed the development of autonomous visual systems in intelligent vehicles, illustrating the broader relevance of reliable visual recognition. LeCun et al. [42] provided a fundamental overview of deep learning and explained why hierarchical representation learning is effective for complex recognition tasks. These studies support the use of CNN-based models in image classification. However, deep models trained directly on raw microscopic images may require large datasets and can be sensitive to background noise, illumination variation, and overfitting.

Despite these advances, several limitations remain in existing studies. Classical computer-vision methods are interpretable and computationally efficient, but they are sensitive to segmentation errors, noise, and handcrafted feature limitations. Contour-based methods capture important boundary information but often lack adaptive learning ability. Fourier descriptors provide compact global shape representation but may be weak in describing local deformation. Wavelet descriptors can capture local and multi-scale variations but require appropriate basis selection and parameter tuning. Shallow machine-learning models depend heavily on manually extracted features, while deep CNN-based models may require large datasets and can be sensitive to background complexity and noise. These limitations motivate the development of a hybrid framework that jointly uses contour geometry, spectral–wavelet descriptors, noise correction, and CNN-based hierarchical learning.

The proposed method addresses these limitations by integrating contour-based point-signal representation, noise suppression, defect-point correction, Fourier and wave-let analysis, and CNN-based classification into a unified framework. Unlike methods that rely only on raw spatial images, the proposed approach first converts micro-object boundaries into point-signal representations, allowing statistical and spectral analysis of con-tour structure. Unlike purely handcrafted contour descriptors, the proposed method uses CNN-based learning to extract hierarchical discriminative features from enriched con-tour-signal representations. Unlike standard CNN-only approaches, it explicitly incorporates global frequency information, local wavelet characteristics, and noise-robust filtering before classification. Therefore, the main contribution is not the individual use of Fourier transform, wavelet transform, or CNN, which are established techniques, but their integrated use in a contour-signal-based microscopic micro-object classification framework. This integration is designed to improve robustness under noise, structural variability, and intra-class similarity, which are key challenges in microscopic imaging.

## 3. Materials and Methods

### 3.1. Problem Formulation

The mathematical expressions used in this section can be divided into two groups. The first group consists of standard equations from digital image processing, contour analysis, signal processing, wavelet analysis, similarity measurement, and supervised deep learning. These include contour-point representation, Fourier transform, wavelet decomposition, cosine similarity, Softmax probability estimation, and cross-entropy loss. These equations are used as established tools and are cited accordingly. The second group consists of framework-specific formulations introduced in this study to describe the proposed processing pipeline, including aggregated contour disturbance modeling, defect-point detection and correction, and fused contour-spectral feature representation. These equations are not intended to introduce new mathematical theory; rather, they formalize how the proposed method combines established components for microscopic pollen-image classification.

The proposed method is formulated based on established principles of digital image processing, contour analysis, signal representation, spectral transformation, wavelet decomposition, and supervised deep-learning classification. In microscopic image analysis, an input image usually contains object shape, boundary, texture, color, illumination, and background information. Classical image-processing theory shows that preprocessing, segmentation, contour extraction, and feature representation are fundamental steps for transforming raw image data into discriminative object descriptors [27]. In addition, contour analysis has been widely used for object recognition because the external boundary of an object provides important geometric and structural information [28]. For microscopic micro-objects such as pollen grains, cells, microorganisms, and mineral particles, the contour often contains class-specific shape characteristics; therefore, contour-based modeling is suitable for reducing background influence and emphasizing object morphology. Let the input microscopic image be defined as:

(1) $$I\in R^{H\times W\times 3}$$

where H and W denote the image height and width, respectively. The objective of micro-object classification is to assign the input image *I* to one of *K* predefined classes:

(2) $$y\in \left\{1,2,\ldots ,K\right\}.$$

The first stage of the proposed method transforms the image into a contour-based representation. After preprocessing and segmentation, the boundary of the target micro-object is extracted and represented as a sequence of contour points:

(3) $$C=\{x_{i}{\}}_{i=1}^{N}$$

where $x_{i}$ denotes the i-th contour point and N is the total number of contour points. This representation follows the principle that an object boundary can be treated as a structured sequence or point signal, allowing the use of statistical and spectral signal-processing techniques for shape analysis [2,16]. Under real microscopic imaging conditions, the extracted contour may be affected by noise, illumination variation, segmentation error, and defective boundary points. Therefore, the observed contour signal can be expressed as:

(4) $$\tilde{C}=C+n$$

where $\tilde{C}$ is the observed noisy contour and η denotes the aggregated disturbance caused by imaging noise and contour extraction errors. In the proposed framework, this disturbance may include Gaussian fluctuation noise, impulse noise, salt-and-pepper noise, and local contour defects. Noise modeling and filtering are therefore included before feature extraction to improve the stability of the contour representation.

To extract discriminative information from the contour signal, the proposed method applies Fourier and wavelet transformations. Fourier analysis provides global frequency information, while wavelet analysis provides local and multi-scale representation of contour variations. These transformations are widely used in image and signal processing for analyzing non-stationary and multi-resolution structures [21,23,24]. The transformed contour representation can be written as:

(5) $$z=\Phi \left(\tilde{C}\right),$$

where *Φ*(⋅) denotes the hybrid feature extraction operator composed of filtering, defect-point correction, Fourier transform, continuous wavelet transform, discrete wavelet transform, and feature fusion.

The final classification is performed using a convolutional neural network. CNNs are suitable for this task because convolutional filters can learn local and hierarchical patterns from structured spatial or transformed feature representations. The classifier is defined as:

(6) $$y=f_{\theta }(z)$$

where $f_{\theta }(\cdot )$ denotes the CNN model with learnable parameters θ, z is the extracted hybrid contour-signal representation, and $\hat{y}$ is the predicted class label. In probabilistic form, the model estimates the posterior class probability:

(7) $$P\left(y=k|z;\theta \right)=\frac{exp(s_{k})}{\sum_{j=1}^{K}exp(s_{j})},$$

where $s_{k}$ is the logit corresponding to the *k*-th class. The CNN is trained by minimizing the categorical cross-entropy loss, which is a standard objective function for supervised multi-class classification. For a training set containing *M* samples, the loss function is defined as:

(8) $$L_{CE}\left(\theta \right)=-\frac{1}{M}\sum_{m=1}^{M}\sum_{k=1}^{K}y_{m,k}log\left({\hat{p}}_{m,k}\right),$$

where $y_{m,k}$ is the one-hot encoded ground-truth label of the m-th sample and ${\hat{p}}_{m,k}$ is the predicted probability for class k. This loss function is not proposed as a new contribution; rather, it is used as an established optimization criterion for training the CNN classifier [34].

Thus, the complete learning objective is:

(9) $$\theta^{*}=arg\underset{\theta }{min}L_{CE}(\theta ).$$

The overall problem can therefore be summarized as learning a robust mapping:

(10) $$I\to C\to \tilde{C}\to z\to \hat{y},$$

where the raw microscopic image is transformed into a contour representation, corrected under noisy conditions, converted into spectral–wavelet features, and classified using a CNN. The contribution of the proposed formulation is not the individual use of contour extraction, Fourier transform, wavelet transform, or cross-entropy loss, since these are established methods, but their integration into a unified contour-signal-based classification framework for microscopic micro-object recognition.

### 3.2. Proposed Framework for Micro-Object Identification

**Figure 1:**
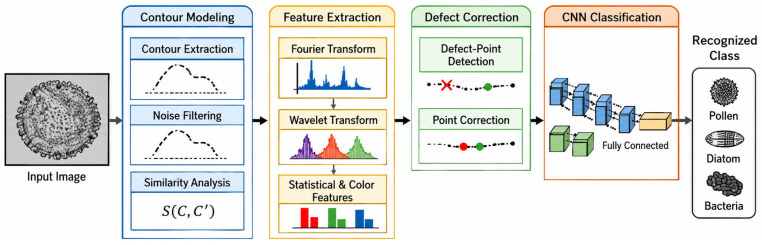
Overall architecture of the proposed microscopic micro-object classification framework.

The proposed framework for microscopic micro-object classification is illustrated in Figure 1. The framework consists of four main processing stages: contour modeling, feature extraction, defect correction, and CNN-based classification. The input microscopic image is first transformed into a contour-based representation. Then, spectral, wavelet, statistical, and color features are extracted from the processed contour signal. After that, defective contour points are detected and corrected. Finally, the corrected and enriched feature representation is classified using a convolutional neural network.

In the first stage, the input microscopic image is processed by the contour modeling module. This module includes three main operations: contour extraction, noise filtering, and similarity analysis. Contour extraction is used to obtain the boundary structure of the micro-object from the original microscopic image. Since the boundary of a micro-object contains important shape information, the extracted contour is represented as a point-based signal. Noise filtering is then applied to suppress distortions caused by imaging noise, background artifacts, and irregular contour points. After filtering, similarity analysis is performed using the similarity measure $S(C,C^{\prime })$, where C denotes the extracted contour and $C^{\prime }$ denotes a reference or normalized contour representation. This step helps evaluate the structural correspondence between the observed contour and reference contour patterns.

In the second stage, the processed contour representation is passed to the feature extraction module. This module extracts complementary descriptors using Fourier transform, wavelet transform, and statistical/color feature analysis. The Fourier transform captures the global frequency structure of the contour signal and describes the overall shape characteristics of the micro-object. The wavelet transform provides multi-scale local analysis and is useful for detecting local contour variations, boundary irregularities, and structural details. In addition, statistical and color features are extracted to describe the intensity distribution, color composition, and morphometric properties of the object. The combination of these features allows the model to represent both global and local characteristics of microscopic micro-objects.

In the third stage, the extracted features are refined using the defect-correction module. This module consists of defect-point detection and point correction. Defective points may appear because of noise, segmentation errors, missing boundary fragments, or local distortions in the contour signal. The defect-point detection step identifies abnormal points that deviate from the expected contour structure. Then, the point-correction step restores these defective regions by adjusting abnormal points according to neighboring valid points. This correction improves the stability of the contour representation and reduces the negative effect of noise on the final classification.

In the final stage, the corrected and enriched representation is provided to the CNN classification module. The convolutional layers learn hierarchical structural patterns from the extracted features, while the fully connected layer performs final decision making. The output layer assigns the input object to one of the predefined microscopic micro-object classes.

In this study, the recognized classes include examples such as pollen, diatom, and bacteria, as shown in Figure 1. Thus, the proposed framework combines contour modeling, spectral–wavelet feature extraction, defect correction, and CNN-based classification in a unified pipeline for robust microscopic micro-object recognition.

#### 3.2.1. Point-Signal Representation of Micro-Object Contours

In microscopic image classification, the boundary structure of a micro-object often contains highly discriminative information. For objects such as pollen grains, microorganisms, cells, and mineral particles, class-specific morphology is reflected not only in the full image texture but also in the external contour, including radial variation, curvature, local irregularity, sharp transitions, and global shape symmetry. Therefore, representing the object boundary as a contour-based point signal provides a compact and informative description of micro-object structure. The use of contour-based point-signal representation is motivated by several factors. First, it reduces the influence of irrelevant background regions. In raw microscopic images, background artifacts, uneven illumination, and acquisition noise may affect the classification process. By extracting the object contour, the analysis focuses on the structural boundary of the target object rather than the entire image. Second, a contour sequence can be interpreted as a structured signal. This makes it possible to apply statistical descriptors, Fourier transform, continuous wavelet transforms, and discrete wavelet transform to analyze both global and local boundary characteristics. Third, after normalization and resampling, contours with different sizes and different numbers of boundary points can be represented in a fixed-length form, enabling direct comparison between micro-objects.

Compared with raw pixel-based representation, the contour-signal representation provides a more shape-oriented description. This is particularly useful when different classes have similar color or texture but differ in boundary morphology. Compared with handcrafted contour descriptors alone, the proposed representation is more flexible because the extracted point signal can be further processed using filtering, defect-point correction, spectral–wavelet decomposition, and CNN-based classification. Thus, the contour-based point signal acts as an intermediate representation that connects geometric shape analysis with learning-based classification.

Several approaches can be used to analyze object boundaries as structured sequences or random time-series representations. Each method has specific advantages and limitations. Table 1 compares commonly used boundary-analysis methods and explains why a hybrid contour-signal representation is selected in this study.

The comparison shows that each boundary-analysis method captures only part of the contour information. Chain-code and radial-distance representations are compact but may be affected by noise or starting-point selection. Curvature descriptors capture local irregularities but are sensitive to small segmentation errors. Fourier descriptors provide global shape information but do not sufficiently represent localized boundary deformation. Wavelet descriptors provide multi-scale analysis but depend on the selected mother wavelet and decomposition level. Statistical random time-series descriptors are interpretable but may not be sufficiently discriminative alone. For this reason, the proposed framework does not rely on a single boundary descriptor. Instead, it uses a hybrid representation in which the normalized contour signal is processed using filtering, defect-point correction, Fourier descriptors, CWT, DWT, and statistical features. The fused representation is then passed to a CNN classifier. This design combines the interpretability of contour-signal analysis with the adaptive learning ability of convolutional neural networks. Therefore, the proposed method is better suited for microscopic micro-object classification under noise, structural variability, and intra-class similarity.

Each micro-object image is converted into a discrete contour representation, which is then modeled as a point signal. Let the extracted contour of the observed micro-object be denoted as Equation (3). Similarly, let the reference contour representation be denoted as:

(11) $$C^{\prime }=\{c_{i}^{\prime }{\}}_{i=1}^{N},$$

where $c_{i}^{\prime }=(x_{i}^{\prime },y_{i}^{\prime })$ is the i-th point of the reference contour. Before similarity computation, both C and $C^{\prime }$ are resampled to the same number of points and normalized to reduce the influence of object size and position.

The centroid of the observed contour C is calculated as:

(12) $$\mu_{C}=\frac{1}{N}\sum_{i=1}^{N}c_{i}.$$

Then, each contour point is centered and normalized as:

(13) $${\hat{c}}_{i}=\frac{c_{i}-\mu_{C}}{\alpha_{C}},$$

where $\alpha_{C}$ is the scale-normalization coefficient defined as:

(14) $$\alpha_{C}=\underset{1\leq i\leq N}{max}∥c_{i}-\mu_{C}{∥}_{2}.$$

In the same way, the reference contour $C^{\prime }$ is normalized as:

(15) $${\hat{c}}_{i}^{\prime }=\frac{c_{i}^{\prime }-\mu_{C^{\prime }}}{\alpha_{C^{\prime }}},$$

where

(16) $$\mu_{C^{\prime }}=\frac{1}{N}\sum_{i=1}^{N}c_{i}^{\prime },$$

and

(17) $$\alpha_{C^{\prime }}=\underset{1\leq i\leq N}{max}∥c_{i}^{\prime }-\mu_{C^{\prime }}{∥}_{2}.$$

After normalization, the contour point sets are converted into vector forms:

(18) $$\hat{C}=[{\hat{c}}_{1},{\hat{c}}_{2},\ldots ,{\hat{c}}_{N}],$$

and

(19) $${\hat{C}}^{\prime }=[{\hat{c}}_{1}^{\prime },{\hat{c}}_{2}^{\prime },\ldots ,{\hat{c}}_{N}^{\prime }].$$

The cosine similarity between the observed contour C and the reference contour $C^{\prime }$ is then defined as:

(20) $$S(C,C^{\prime })=\frac{\left\langle \hat{C},{\hat{C}}^{\prime }\right\rangle }{∥\hat{C}{∥}_{2}∥{\hat{C}}^{\prime }{∥}_{2}},$$

where $\left\langle \hat{C},{\hat{C}}^{\prime }\right\rangle$ denotes the scalar product of the normalized contour vectors, and $∥\hat{C}{∥}_{2}$ and $∥{\hat{C}}^{\prime }{∥}_{2}$ are their Euclidean norms.

The value of $S(C,C^{\prime })$ indicates the normalized structural similarity between two contour representations. A larger value of $S(C,C^{\prime })$ means that the observed contour is more like the reference contour. Since the contours are normalized before comparison, the similarity coefficient becomes less sensitive to absolute object size and contour magnitude. This property is important in microscopic imaging because objects belonging to the same class may appear with different scales, boundary lengths, and orientations.

For comparison, the Euclidean distance between the normalized contours can also be written as:

(21) $$D\left(C,C^{\prime }\right)=\;∥\hat{C}-{\hat{C}}^{\prime }{∥}_{2}.$$

However, $D(C,C^{\prime })$ measures absolute geometric deviation, whereas $S(C,C^{\prime })$ measures normalized directional similarity between contour representations. Therefore, cosine similarity is more suitable as an auxiliary descriptor when the objective is to compare the shape pattern of normalized micro-object contours rather than their absolute magnitude. In the proposed framework, $S(C,C^{\prime })$ is not used as an independent classifier. Instead, it is included as an additional similarity descriptor together with statistical, spectral, Fourier, and wavelet-based features. The fused representation is then passed to the CNN classifier for final micro-object classification.

#### 3.2.2. Noise Modeling and Signal Distortion

In practical microscopic imaging conditions, contour signals may be affected by noise and local boundary distortions, which makes reliable identification more difficult. In this study, the filtering strategy is selected according to the behavior of the disturbance in the contour signal. Gaussian-type fluctuation noise is treated as a continuous perturbation that affects neighboring contour samples, whereas impulse and salt-and-pepper noise appear as isolated abnormal values or abrupt local discontinuities. In addition, segmentation errors may introduce missing, displaced, or defective contour points.

Let the observed contour signal be expressed as:

(22) $$\tilde{C}(n)=C(n)+\eta (n),$$

where C(n) is the ideal contour signal, $\tilde{C}(n)$ is the observed noisy contour signal, and η(n) denotes the total disturbance. In practice, η(n) may include continuous fluctuation noise, impulse noise, salt-and-pepper artifacts, and contour defects. Therefore, the disturbance can be represented as:

(23) $$\eta (n)=\eta_{G}(n)+\eta_{I}(n)+\eta_{S}(n)+\eta_{D}(n),$$

where $\eta_{G}(n)$ denotes Gaussian-type fluctuation noise, $\eta_{I}(n)$ denotes impulse noise, $\eta_{S}(n)$ denotes salt-and-pepper noise, and $\eta_{D}(n)$ denotes local contour defects caused by segmentation or boundary-extraction errors.

Gaussian filtering is used to reduce continuous fluctuation noise. It performs local weighted averaging and is suitable when neighboring contour samples are affected by smooth random perturbations. The Gaussian-filtered contour signal is computed as:

(24) $$C_{G}(n)=\sum_{m=-r}^{r}G(m)\tilde{C}(n-m),$$

where G(m) is the Gaussian kernel and r is the window radius. This operation reduces high-frequency fluctuation while preserving the general contour trend.

Median filtering is used to suppress impulse and salt-and-pepper noise. These noise types usually appear as isolated abnormal values in the contour signal. The median-filtered signal is defined as:

(25) $$C_{M}(n)=median\{\tilde{C}(n-r),\ldots ,\tilde{C}(n),\ldots ,\tilde{C}(n+r)\}.$$

Compared with linear averaging, median filtering is more robust to outliers and is therefore suitable for correcting sudden abnormal contour samples.

Contour-aware filtering is used to reduce noise while preserving important boundary geometry. A standard smoothing filter may remove useful local contour details together with noise. Therefore, the contour-aware filter considers local contour consistency and gives higher importance to neighboring points that preserve the boundary structure. It can be written as:

(26) $$C_{A}(n)=\frac{\sum_{m\in \Omega_{n}}w(n,m)C_{M}(m)}{\sum_{m\in \Omega_{n}}w(n,m)},$$

where $\Omega_{n}$ is the local neighborhood of point n, and w(n,m) is a contour-dependent weight. The weight is larger when neighboring points have similar contour direction, curvature, or local structural behavior. This helps reduce noise without excessively smoothing sharp boundary transitions.

After filtering, defective contour points are detected and corrected. Two criteria are used: local deviation and local gradient magnitude. First, the local mean or median in the neighborhood $\Omega_{n}$ is calculated as:

(27) $${\overset{-}{C}}_{\Omega }(n)=\frac{1}{|\Omega_{n}|}\sum_{m\in \Omega_{n}}C_{A}(m).$$

The local deviation is then defined as:

(28) $$d(n)=|C_{A}(n)-{\overset{-}{C}}_{\Omega }(n)|.$$

The local gradient magnitude is calculated as:

(29) $$g(n)=|C_{A}(n)-C_{A}(n-1)|.$$

A contour point is considered defective if $\;d(n)>\tau_{d}\;\mathrm{or}\;g(n)>\tau_{g},$ where $\tau_{d}$ and $\tau_{g}$ are the deviation and gradient thresholds, respectively. This rule is proposed as a practical defect-point detection criterion for the present framework. It is motivated by the observation that abnormal contour samples usually appear either as points that deviate strongly from their local neighborhood or as abrupt local jumps in the contour signal. Therefore, both local deviation and local gradient magnitude are used to identify defective contour points before Fourier and wavelet feature extraction.

The corrected contour signal is obtained as:

(30) $$C_{F}(n)=\left\{\begin{array}{cc} interp(C_{A}(n-1),C_{A}(n+1)), & \mathrm{if}\;n\;\mathrm{is}\;\mathrm{defective},\\ C_{A}(n), & \mathrm{otherwise}. \end{array}\right.$$

Here, interp(⋅) denotes interpolation using adjacent valid contour samples. This correction step is important because Fourier and wavelet descriptors are sensitive to abnormal contour spikes. If defective points are not corrected, they may introduce artificial high-frequency components and reduce classification stability. Thus, Gaussian filtering, median filtering, contour-aware filtering, and defect-point correction are selected according to the expected noise behavior in microscopic contour signals. Gaussian filtering reduces distributed fluctuation noise, median filtering removes isolated impulse-like disturbances, contour-aware filtering preserves boundary geometry, and defect-point correction handles abnormal contour samples caused by segmentation or boundary extraction errors (Table 2).

#### 3.2.3. Feature Extraction from Point Signals

At this stage, informative descriptors are extracted from the contour signal. Basic statistical features include the mean, variance, and standard deviation, which characterize the global properties of the contour representation.

(31) $$\Delta I=|I\left(x_{i}\right)-I\left(x_{j}\right)|$$

This measure captures fine structural differences between neighboring points and helps identify boundaries and local irregularities. Each contour point is also described using its RGB components.

(32) $$x_{i}=\left(R_{i},G_{i},B_{i}\right)$$

These values characterize the color and illumination properties of the micro-object.

Constraining these values improves robustness to geometric transformations such as scaling and rotation. The contour is then modeled as a random time series with the corresponding statistical parameters, assuming local stationarity implies correlation between neighboring contour points, which in turn improves filtering and feature extraction.

#### 3.2.4. Hybrid Spectral–Wavelet Transformation

After contour extraction, normalization, and filtering, the micro-object boundary is represented as a discrete contour signal. Let the normalized and corrected contour signal be denoted as:

(33) C(n),n=1,2,…,N,

where N is the number of sampled contour points. The purpose of the spectral–wavelet transformation is to extract complementary descriptors from C(n). Fourier transform is used to describe the global frequency structure of the contour, while wavelet transforms are used to analyze local and multi-scale variations of the boundary.

The discrete Fourier transform of the contour signal C(n) is defined as:

(34) $$F\left(k\right)=\sum_{n=0}^{N-1}C\left(n\right)\exp\left(-j\frac{2\pi kn}{N}\right),\;\;k=0,1,\ldots ,N-1,$$

where F(k) represents the frequency coefficient corresponding to the k-th frequency component. Fourier descriptors are useful for capturing the global shape characteristics of the contour. However, Fourier transform does not explicitly preserve local positional information. Therefore, wavelet analysis is additionally used to capture localized boundary changes. This is the standard discrete Fourier transform applied to the resampled contour signal. It is used to extract the global frequency characteristics of the pollen boundary. Low-frequency coefficients describe the overall shape, while high-frequency coefficients mainly correspond to small local fluctuations and noise. The continuous wavelet transform of C(n) is defined as:

(35) $$W_{C}(a,b)=\frac{1}{\sqrt{|a|}}\sum_{n=0}^{N-1}C(n)\psi^{*}\left(\frac{n-b}{a}\right),$$

where a is the scale parameter, b is the translation parameter, ψ(⋅) is the mother wavelet, and $\psi^{*}(\cdot )$ denotes its complex conjugate. The coefficient $W_{C}(a,b)$ measures the similarity between the contour signal C(n) and the shifted-scaled wavelet function. Small values of a capture fine local boundary changes, while large values of a describe broader structural variations of the contour.

For efficient hierarchical representation, the discrete wavelet transform is also applied. In DWT, the contour signal is decomposed into approximation and detail components:

(36) $$C(n)=A_{J}(n)+\sum_{j=1}^{J}D_{j}(n),$$

where $A_{J}(n)$ is the approximation component at decomposition level J, and $D_{j}(n)$ is the detail component at level j. The approximation component represents the low-frequency global shape of the contour, while the detail components represent high-frequency local changes, including boundary irregularities, sharp transitions, and possible residual noise.

The DWT coefficients are computed using low-pass and high-pass filtering followed by downsampling:

(37) $$A_{j}(m)=\sum_{n}h(n-2m)A_{j-1}(n),$$

(38) $$D_{j}(m)=\sum_{n}g(n-2m)A_{j-1}(n),$$

where h(⋅) is the low-pass filter, g(⋅) is the high-pass filter, $A_{j}(m)$ is the approximation coefficient at level j, and $D_{j}(m)$ is the corresponding detail coefficient. At the first level,

(39) $$A_{0}(n)=C(n).$$

In this study, Haar and Daubechies wavelets are used because they provide complementary descriptions of contour structure. The Haar wavelet is defined as:

(40) $$\psi_{H}(t)=\left\{\begin{array}{cc} 1, & 0\leq t<\frac{1}{2},\\ -1, & \frac{1}{2}\leq t<1,\\ 0, & \mathrm{otherwise}. \end{array}\right.$$

The Haar wavelet is simple, fast, and sensitive to abrupt changes. Therefore, it is suitable for detecting sharp contour transitions, sudden boundary irregularities, and local discontinuities in microscopic object boundaries.

Daubechies wavelets are compactly supported orthogonal wavelets with higher-order smoothness. In this study, Db5 and Db8 are used to represent smoother contour variations. Compared with Haar, Daubechies wavelets provide better approximation of gradual structural changes and reduce excessive sensitivity to small local fluctuations. Therefore, Db5 and Db8 are suitable for micro-objects whose boundaries contain smooth but discriminative shape patterns.

**Figure 2:**
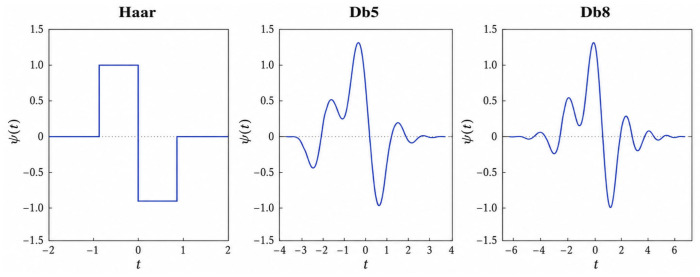
Visual comparison of the selected wavelet mothers used in the proposed framework.

To better illustrate the differences between the selected wavelet mothers, Figure 2 shows representative shapes of the Haar, Db5, and Db8 wavelets. Although all three wavelets are suitable for multi-scale contour analysis, they emphasize different signal characteristics. Haar has a discontinuous, piecewise-constant structure and is highly sensitive to abrupt local signal changes. Db5 provides an intermediate level of smoothness and is useful for capturing both local detail and moderately smooth contour variations. Db8 has a smoother structure and longer support, making it more suitable for representing gradual structural variations and low-frequency contour trends.

The Haar wavelet is piecewise constant and suitable for abrupt contour transitions. Db5 provides balanced multi-scale detail, while Db8 better represents smoother low-frequency contour trends. The selected wavelet coefficients are combined with Fourier and statistical descriptors to construct the hybrid feature vector:

(41) $$Z=\left[F,W_{C},A_{J},D_{1},D_{2},\ldots ,D_{J},Q\right],\;$$

where F denotes Fourier descriptors, $W_{C}$ denotes CWT-based descriptors, $A_{J}$ and $D_{j}$ denote DWT approximation and detail coefficients, and Q denotes statistical contour descriptors such as mean, variance, standard deviation, skewness, kurtosis, and energy.

Thus, the use of Haar, Db5, and Db8 wavelets is motivated by their complementary properties. Haar captures sharp and abrupt boundary changes, while Db5 and Db8 capture smoother multi-scale structural variations. The resulting spectral–wavelet representation provides richer contour information than using Fourier descriptors or raw contour points alone.

#### 3.2.5. Defect-Point Detection and Correction

Noise and structural distortions often introduce defective points into the contour. These points are detected and corrected using threshold-based procedures. Hard thresholding is first applied as follows:

(42) $$x=\left\{\begin{array}{cc} x, & |x|>T\\ 0, & |x|\leq T \end{array}\right.$$

Soft thresholding is then used as a smoother alternative:

(43) $$x=\mathrm{sign}\left(x\right)(|x|-T)$$

The threshold T value is selected by minimizing the mean-squared error (MSE). Together, these operations suppress noise while preserving informative signal components.

#### 3.2.6. Hybrid Spline–Wavelet CNN Model

After preprocessing and feature extraction, the processed representations are passed to a hybrid neural architecture. Hybrid feature construction: The signal representation is enriched using spline interpolation, Daubechies wavelets (Db5 and Db8), and Fourier components. These fusions capture complementary information from multiple domains. CNN-based classification: Final recognition is performed using a CNN composed of convolutional layers for feature extraction, pooling layers for dimensionality reduction, and fully connected layers for decision making. Operating on the enriched representation improves robustness and classification accuracy.

#### 3.2.7. Proposed Algorithm and Computational Complexity

The complete procedure of the proposed method is summarized in Algorithm 1. Compared with the previous high-level description, the revised algorithm provides a more explicit implementation workflow, including contour construction, signal processing, feature fusion, and CNN-based classification.
**Algorithm 1.** Micro-Object Identification and Classification Using Contour-Signal, Wavelet, and CNN Features**Input:** Microscopic image $I\in R^{H\times W\times C}$, number of contour points N, number of classes K, trained CNN parameters θ.
**Output:** Predicted class label $\hat{y}$. Image preprocessing1.1. Read the input microscopic image I.1.2. Normalize the pixel intensities of I.1.3. Apply contrast adjustment to improve object–background separability.1.4. Apply optional smoothing to reduce weak acquisition noise.
2.Object segmentation and contour extraction2.1. Segment the target micro-object from the background.2.2. Extract the external object contour:$C=\{c_{i}{\}}_{i=1}^{M},$where $c_{i}=(x_{i},y_{i})$ is the i-th contour point and M is the original number of contour points.
3.Contour normalization and resampling3.1. Compute the contour centroid:$\mu_{C}=\frac{1}{M}\sum_{i=1}^{M}c_{i}.$3.2. Compute the scale coefficient:$\alpha_{C}=\underset{1\leq i\leq M}{max}∥c_{i}-\mu_{C}{∥}_{2}.$3.3. Normalize the contour points:${\hat{c}}_{i}=\frac{c_{i}-\mu_{C}}{\alpha_{C}}.$3.4. Resample the normalized contour to a fixed number of points N:$\hat{C}=\{{\hat{c}}_{n}{\}}_{n=1}^{N}.$
4.Point-signal construction4.1. Convert the normalized contour into a contour signal:C(n),n=1,2,…,N.4.2. Construct signal descriptors using coordinate, radial-distance, curvature, or intensity-based values.
5.Noise modeling and filtering5.1. Model the observed contour signal as:$\tilde{C}(n)=C(n)+\eta (n),$where η(n) denotes contour disturbance caused by noise or segmentation defects. This equation is a framework-specific formulation used to describe how microscopic imaging noise and contour-extraction errors affect the ideal contour signal. It follows the common additive-noise assumption in signal and image processing, but here the disturbance term is interpreted specifically in the context of contour signals extracted from microscopic pollen images.5.2. Apply Gaussian filtering to reduce continuous fluctuation noise.5.3. Apply median filtering to reduce impulse and salt-and-pepper noise.5.4. Apply contour-aware filtering to preserve boundary geometry.
6.Defect-point detection and correction6.1. Detect abnormal contour points using threshold-based deviation analysis:$|\tilde{C}(n)-{\overset{-}{C}}_{w}(n)|>\tau ,$where ${\overset{-}{C}}_{w}(n)$ is the local mean in a sliding window and τ is the defect threshold.6.2. Correct each detected defective point using neighboring valid contour points.6.3. Obtain the corrected contour signal $C_{f}(n)$.
7.Similarity descriptor computation7.1. Compare the corrected contour $C_{f}$ with the reference contour $C^{\prime }$.7.2. Compute the cosine similarity:$S(C_{f},C^{\prime })=\frac{\left\langle C_{f},C^{\prime }\right\rangle }{∥C_{f}{∥}_{2}∥C^{\prime }{∥}_{2}}.$
8.Spectral and wavelet feature extraction8.1. Compute the Fourier descriptors:$F\left(k\right)=\sum_{n=0}^{N-1}C_{f}\left(n\right)\exp\left(-j\frac{2\mathrm{\pi kn}}{N}\right).$8.2. Compute the continuous wavelet coefficients:$W_{C}\left(a,b\right)=\frac{1}{\sqrt{|a|}}\sum_{n=0}^{N-1}C_{f}\left(n\right)\psi^{*}\left(\frac{n-b}{a}\right).$8.3. Compute the DWT approximation and detail coefficients:$C_{f}\left(n\right)=A_{J}\left(n\right)+\sum_{j=1}^{J}D_{j}\left(n\right).$
9.Feature fusion9.1. Compute statistical descriptors Q, including mean, variance, standard deviation, energy, skewness, and kurtosis.9.2. Construct the final hybrid feature vector:$Z=[S(C_{f},C^{\prime }),F,W_{C},A_{J},D_{1},D_{2},\ldots ,D_{J},Q].$
10.CNN classification10.1. Feed Z into the trained CNN classifier $f_{\theta }\left(\cdot \right)$.10.2. Compute the class probability vector:$p=f_{\theta }\left(Z\right).$10.3. Determine the predicted class label:$\hat{y}=\arg\underset{k\in \{1,\ldots ,K\}}{max}p_{k}.$
11.Return $\hat{y}$.

The computational complexity of the proposed framework depends on the image size, the number of contour points, the number of wavelet decomposition levels, and the CNN architecture. Let the input image size be H×W, the number of resampled contour points be N, the number of wavelet decomposition levels be J, and the number of CNN layers be L.

Image preprocessing and segmentation are performed on the full image and require approximately O(HW). Contour extraction also depends on the image size and can be approximated as O(HW).

After extracting the contour, centroid calculation, scale normalization, and resampling require linear complexity with respect to the number of contour points, and noise filtering and defect-point correction are also linear when a fixed-size sliding window is used O(N).

The Fourier transform requires O(NlogN) when implemented using the fast Fourier transform. The discrete wavelet transform requires approximately O(JN), which becomes O(N) when the number of decomposition levels J is fixed. The continuous wavelet transform is more expensive because it evaluates the signal over multiple scales. If A scales are used, its complexity is approximately O(AN).

The CNN inference complexity can be written as:

(44) $$O\left(\sum_{l=1}^{L}H_{l}W_{l}K_{l}^{2}C_{l-1}C_{l}\right),$$

where $H_{l}$ and $W_{l}$ are the spatial dimensions of the feature map at layer l, $K_{l}$ is the kernel size, $C_{l-1}$ is the number of input channels, and $C_{l}$ is the number of output channels.

Therefore, the total computational complexity of the proposed framework can be approximated as:

(45) $$O\left(HW+NlogN+AN+\sum_{l=1}^{L}H_{l}W_{l}K_{l}^{2}C_{l-1}C_{l}\right).$$

Since N is fixed after contour resampling and the CNN architecture is lightweight, the proposed method remains computationally feasible for microscopic micro-object classification. The most computationally expensive stages are CWT computation and CNN inference, while contour normalization, filtering, defect correction, and DWT decomposition are linear with respect to the number of contour points.

### 3.3. Implementation Details and Intermediate Feature Dimensions

To facilitate reproducibility, the complete data flow together with the dimensionality of the intermediate representations is explicitly described in this subsection. Unlike the conceptual framework illustrated in Figure 1, the following description specifies the actual data representation passed between consecutive processing modules. The input to the proposed framework is an RGB microscopic image with spatial dimensions H×W×3. During preprocessing, the image dimensions remain unchanged, while Gaussian and median filtering suppress sensor noise, impulse-like artifacts, and illumination-related fluctuations. The filtered image is subsequently segmented to obtain a binary mask representing the target microscopic object.

Boundary extraction converts the segmented object into an ordered contour consisting of N boundary points using Equation (11). This equation follows the standard boundary representation used in contour-based image analysis, where the object boundary is represented as an ordered sequence of two-dimensional coordinates. It is introduced because pollen grains are primarily characterized by boundary morphology, and converting the segmented object into contour points allows subsequent signal-processing analysis.

Since different microscopic objects produce contours with different numbers of boundary samples, direct comparison is not possible. Therefore, all contours are resampled using linear interpolation to obtain a fixed-length representation containing $N_{r}$ equally spaced contour points:

(46) $$C_{r}=\{(x_{i},y_{i}){\}}_{i=1}^{N_{r}}.$$

The normalized contour is subsequently converted into a one-dimensional radial-distance signal:

(47) $$S=\{r_{1},r_{2},\ldots ,r_{N_{r}}\},$$

where each sample $r_{i}$ represents the Euclidean distance between the i-th contour point and the contour centroid. This representation serves as the input to the filtering and defect-correction modules. The radial-distance signal is introduced to convert the two-dimensional normalized contour into a one-dimensional structured signal. This formulation is motivated by the fact that shape variations of pollen grains can be represented by changes in the distance between boundary points and the centroid. Therefore, the contour can be analyzed using statistical, Fourier, and wavelet-based signal descriptors.

Adaptive Gaussian filtering, median filtering, contour-aware filtering, and defect-point correction preserve the dimensionality of the contour signal. Consequently, the filtered and corrected contour signal remains:

(48) $$S_{F}\in R^{N_{r}\times 1}.$$

The filtered contour signal is then analyzed using complementary spectral transforms. The fast Fourier transform converts the contour signal into the frequency domain:

(49) $$S_{FFT}\in R^{N_{f}},$$

where only the first $N_{f}$ low-frequency coefficients are retained because higher-frequency coefficients mainly describe noise and insignificant contour fluctuations.

The continuous wavelet transform computes a two-dimensional time-scale representation:

(50) $$S_{CWT}\in R^{N_{r}\times S},$$

where S denotes the number of analyzed wavelet scales. If dimensionality reduction or coefficient selection is applied, the selected CWT descriptor is represented as:

(51) $$F_{CWT}\in R^{N_{c}}.$$

Similarly, the discrete wavelet transform produces:

(52) $$S_{DWT}\in R^{N_{d}},$$

where $N_{d}$ denotes the number of retained approximation and detail coefficients.

The final descriptor is obtained by concatenating all extracted features:

(53) $$Z=[F_{stat},F_{color},S_{FFT},F_{CWT},S_{DWT},F_{sim}],$$

where $F_{stat}\in R^{m}$ denotes the statistical descriptor vector, $F_{color}\in R^{c}$ denotes color descriptors, $S_{FFT}\in R^{N_{f}}$ denotes retained Fourier coefficients, $F_{CWT}\in R^{N_{c}}$ denotes selected CWT descriptors, $S_{DWT}\in R^{N_{d}}$ denotes DWT descriptors, and $F_{sim}\in R^{1}$ denotes the cosine-similarity descriptor. This equation is proposed to formalize the feature-fusion strategy used in the framework. The fused vector combines statistical, color, Fourier, wavelet, and similarity descriptors so that the CNN receives complementary global, local, multi-scale, and structural information. This fusion is motivated by the limitation of using any single descriptor alone. Therefore, the fused feature dimension is:

(54) $$D=m+c+N_{f}+N_{c}+N_{d}+1.$$

The resulting fused-feature vector $Z\in R^{D}\;$ serves as the input to the CNN classifier. The CNN maps this descriptor to posterior class probabilities $p=f_{\theta }\left(Z\right),\;p\in R^{K},$ where K is the number of micro-object classes and $f_{\theta }(\cdot )$ denotes the CNN model with trainable parameters θ.

Unlike conventional CNN approaches that directly process raw microscopic images, the proposed CNN operates on enriched contour-signal representations. Consequently, the convolutional layers learn hierarchical relationships among spectral, statistical, color, and structural descriptors instead of raw pixel intensities, improving robustness under noisy microscopic imaging conditions (Table 3).

The classification stage used a compact CNN architecture tailored to hybrid contour-signal representations. The network contains three convolutional blocks with 32, 64, and 128 filters, respectively. Each block includes convolution, batch normalization, ReLU activation, and max pooling. After hierarchical feature extraction, the resulting feature maps are aggregated using global average pooling (or flattening), followed by a fully connected layer with 128 neurons and a dropout rate of 0.5. The final classification layer uses Softmax activation to predict the probability distribution over the target micro-object classes. This lightweight design was chosen to balance discriminative power with robustness on limited microscopic datasets.

The classification stage uses a CNN-based classifier because the proposed hybrid representation preserves local and multi-scale structural patterns of microscopic contours. After contour extraction, filtering, Fourier analysis, and wavelet decomposition, the resulting representation contains local boundary variations, frequency-domain descriptors, and hierarchical multi-scale features. Convolutional filters are suitable for such data because they can learn local dependencies and progressively construct higher-level discriminative representations. A lightweight CNN was selected instead of a very deep architecture for three reasons. First, the microscopic pollen dataset used in this study is moderate in size; therefore, very deep models may increase the risk of overfitting. Second, the proposed feature-extraction pipeline already provides informative contour-signal, spectral, wavelet, and statistical descriptors, reducing the need for excessively deep feature extraction. Third, a compact CNN provides a better balance between classification accuracy, training cost, and inference efficiency, which is important for practical microscopic-image classification systems.

The selected CNN is not intended to exclude other learning models. Therefore, in the experimental section, the proposed method is compared with both shallow machine-learning methods and deep-learning models to justify the classifier choice.

The CNN was trained with the Adam optimizer using a learning rate of 0.001, a batch size of 16–32, and 50–100 training epochs. Cross-entropy loss was used as the optimization objective. The architecture was intentionally kept lightweight to remain compatible with transformed contour-signal inputs while reducing the risk of overfitting. The implementation was carried out using Python 3.12 framework.

## 4. Results and Discussion

**Figure 3:**
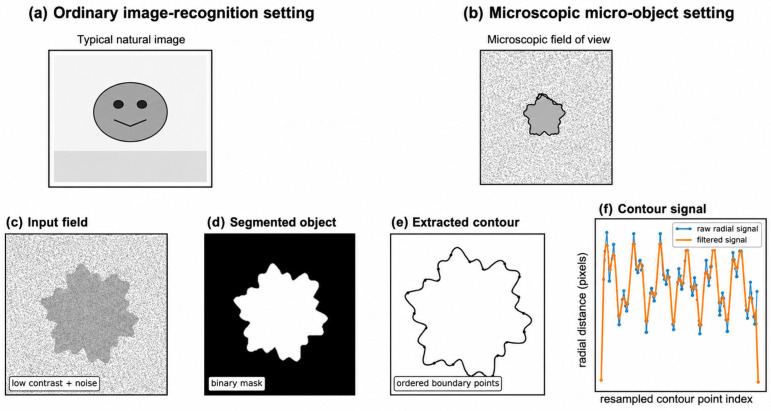
Difference between ordinary image recognition and microscopic micro-object recognition.

To illustrate the visual characteristics of the dataset, representative sample images from the microscopic micro-object classes are shown in Figure 3. These examples demonstrate that microscopic micro-object recognition differs substantially from ordinary image-recognition tasks. In ordinary image recognition, target objects usually occupy a large image region and contain rich semantic cues such as color, texture, object parts, and contextual background. In contrast, microscopic micro-objects are usually small, weakly textured, low-contrast, and strongly affected by acquisition noise, optical blur, illumination variation, and segmentation uncertainty.

As shown in Figure 3, the discriminative information of microscopic micro-objects is concentrated mainly along the object boundary. Different classes may have very similar global appearance, while the most useful class-specific information appears in subtle contour morphology, local boundary irregularities, radial variation, and fine structural patterns. In addition, objects from the same class may show different orientations, sizes, deformations, and noise levels. These characteristics make direct raw-image classification difficult and motivate the proposed contour-signal-based framework. By converting the object boundary into a normalized point-signal representation, the proposed method focuses on morphology-related information and reduces the influence of irrelevant background regions.

Ordinary image-recognition tasks often rely on large target regions, semantic object parts, background context, and rich texture. In contrast, microscopic micro-object recognition is characterized by small target regions, weak contrast, background noise, and subtle contour irregularities. The lower panels illustrate the proposed motivation: microscopic images are segmented, converted into ordered contours, and represented as radial-distance contour signals for subsequent filtering, spectral–wavelet analysis, and CNN-based classification.

To evaluate the proposed hybrid contour-signal and deep-learning framework, experiments were conducted on a microscopic pollen-image dataset containing seven classes: Artemisia, Chenopodiaceae, Cupressaceae, Poaceae, Moraceae, Pinaceae, and Salix. The dataset comprises approximately 4200 images with a class-balanced distribution. The dataset comprises 4200 images with a balanced class distribution, as shown in Table 4. All images were preprocessed and converted into contour-based point-signal representations before feature extraction and classification. Care was taken to avoid overlap between the training and test subsets. The class-wise distribution of the dataset is shown in Table 5. The dataset is approximately balanced, with 600 images per class. Maintaining a balanced distribution is important because it reduces the risk of classifier bias toward dominant classes and supports more reliable interpretation of accuracy and F1-score.

Although the proposed framework is motivated by general challenges in microscopic object recognition, the experimental validation in this study is limited to a seven-class microscopic pollen-image dataset. Therefore, the obtained results should be interpreted specifically in the context of pollen-image recognition rather than as evidence of universal applicability to all tiny micro-objects. Pollen grains provide a useful test case because they contain subtle boundary morphology, weak texture, contour irregularities, and intra-class variability. However, they do not fully represent the morphological diversity of other microscopic objects such as nanoparticles, fibers, rods, cylindrical particles, triangular particles, or chemically synthesized microstructures. These objects may exhibit different shape distributions, surface textures, imaging artifacts, and contour characteristics. Consequently, additional validation on larger and more diverse microscopic object datasets is required before claiming general applicability across all micro-object categories.

The dataset publisher did not provide an official predefined training, validation, and test split. Therefore, in the main experiment, the dataset was divided using a stratified sampling strategy to preserve the class distribution in each subset. Stratified splitting was used because the dataset contains multiple pollen classes, and preserving the same class proportion in the training, validation, and test sets is necessary for fair performance evaluation. However, a single train, validation, and test split may still introduce split-dependent bias. To reduce this risk and to verify the stability of the proposed method, an additional stratified 5-fold cross-validation experiment was conducted. In this protocol, the complete dataset was divided into five folds with approximately equal class distributions. For each run, four folds were used for training and one fold was used for testing. The process was repeated five times so that each fold was used once as the test set. The final performance was reported as the mean and standard deviation across the five folds.

This cross-validation protocol was used to evaluate whether the proposed method remains stable under different data partitions and to ensure that the reported performance is not dependent on a single favorable split.

As shown in Table 6, the proposed method achieved stable performance across all five folds. The mean accuracy was 0.977 ± 0.003, and the mean F1-score was 0.966 ± 0.003. The low standard deviation indicates that the performance is not strongly dependent on a particular train/test partition. These results confirm that the proposed contour-signal, spectral–wavelet, and CNN-based framework generalizes consistently across different stratified data splits and reduces the possibility of training-split bias.

To ensure reproducibility, the Classical Computer-Vision System (Classical CVS) baseline was explicitly defined. The Classical CVS pipeline consists of image preprocessing, object segmentation, contour extraction, handcrafted feature extraction, feature normalization, and shallow classification. The extracted handcrafted features include shape descriptors, contour descriptors, texture descriptors, color/intensity statistics, and HOG features. Shape descriptors include area, perimeter, circularity, eccentricity, compactness, major-axis length, minor-axis length, and aspect ratio. Contour descriptors include radial-distance statistics, curvature statistics, and Fourier contour descriptors. Texture descriptors are extracted using gray-level co-occurrence matrix (GLCM) features, including contrast, correlation, energy, and homogeneity. Color and intensity descriptors include mean, standard deviation, skewness, kurtosis, and histogram-based statistics. The final handcrafted feature vector is normalized using z-score normalization and classified using an SVM classifier with a radial basis function (RBF) kernel. The Classical CVS baseline is therefore defined as:

I→preprocessing→segmentation→contour extraction→handcrafted features→SVM-RBF classification.

This definition distinguishes Classical CVS from the proposed method. The Classical CVS baseline relies on fixed handcrafted descriptors and a shallow classifier, whereas the proposed method combines contour-signal modeling, FT/CWT/DWT descriptors, filtering, defect-point correction, and CNN-based hierarchical learning.

To validate the effectiveness of the proposed method, we compared it with representative existing approaches for small-object, micro-object, contour-based, and structure-aware image recognition. The compared methods were selected according to the methodological categories discussed in the Introduction and Related Work, including classical handcrafted feature extraction, contour-based structural descriptors, spectral descriptors, wavelet-based descriptors, shallow machine-learning classifiers, and CNN-based deep-learning models.

The purpose of this comparison is to determine whether the proposed integrated framework provides a performance advantage over existing individual or partial recognition strategies. In particular, the proposed method is compared with approaches that use only raw spatial image information, only handcrafted structural features, only Fourier descriptors, only wavelet descriptors, and standard CNN-based feature learning. This comparison is important because the proposed method integrates contour-signal modeling, noise suppression, defect-point correction, Fourier analysis, wavelet decomposition, and CNN-based hierarchical classification into a unified framework.

The compared methods are summarized as follows:

1. Classical Computer-Vision pipeline. This baseline follows a conventional recognition strategy consisting of preprocessing, segmentation, handcrafted feature extraction, and classification. The extracted features include shape, contour, intensity, color, and texture descriptors.
2. Contour, statistical descriptor method. This method represents each pollen object using contour-derived statistical descriptors, including mean radial distance, variance, standard deviation, energy, curvature variation, and local boundary deviation. These features are classified using a shallow classifier.
3. Fourier descriptor-based method. This method applies Fourier transform to the normalized contour signal and uses retained low-frequency Fourier coefficients as global shape descriptors. This baseline evaluates the contribution of global spectral contour information.
4. Wavelet descriptor-based method. This method extracts multi-scale contour descriptors using continuous and/or discrete wavelet transform. It evaluates the usefulness of local and multi-scale boundary information without CNN-based hierarchical learning.
5. Shallow machine-learning models. Support Vector Machine, Random Forest, and k-Nearest Neighbor classifiers are trained using handcrafted structural, statistical, spectral, and wavelet descriptors. These models represent traditional intelligent recognition approaches.
6. CNN trained on raw microscopic images. A baseline CNN is trained directly on raw microscopic images. This comparison evaluates whether direct pixel-based deep learning is sufficient for microscopic pollen-image classification.
7. Proposed method. The proposed framework uses contour-signal representation, Gaussian/median/contour-aware filtering, defect-point correction, Fourier transform, continuous wavelet transform, discrete wavelet transform, feature fusion, and CNN-based classification.

The comparative results are presented in Table 7.

As shown in Table 7, the proposed method achieved the highest accuracy and F1-score among all compared methods. The classical CVS and contour, statistical methods were less effective because they rely on handcrafted descriptors and are sensitive to noise, segmentation errors, and boundary deformation. Fourier descriptors improved global shape representation, while wavelet descriptors improved local and multi-scale contour analysis; however, these methods still lacked adaptive hierarchical feature learning. CNN-only and ResNet-50 performed better than traditional baselines, but they mainly depend on raw image appearance and do not explicitly exploit contour-specific spectral–wavelet information. In contrast, the proposed framework combines contour-signal representation, filtering, defect-point correction, FT, CWT, DWT, and CNN-based classification. This integrated design provides more robust and discriminative features for microscopic pollen-image recognition.

Deep-learning baselines were evaluated separately to compare the proposed hybrid CNN framework with standard neural architectures. These models include CNN-only, MobileNetV2, EfficientNet-B0, ResNet-50, and Vision Transformer. The CNN-only model evaluates the contribution of convolutional learning without the full spectral–wavelet feature enhancement, while MobileNetV2, EfficientNet-B0, ResNet-50, and Vision Transformer provide broader comparison with modern deep-learning architectures (Table 8).

The shallow-method comparison shows that conventional classifiers benefit from contour and spectral–wavelet descriptors, but their performance remains limited because they rely on fixed handcrafted representations. Among shallow methods, MLP achieved the strongest performance, but it still does not explicitly model local dependencies in the transformed contour-signal representation. The deep-learning comparison shows that modern neural architecture provides stronger performance than shallow models. However, models trained primarily on raw image appearance remain less effective than the proposed hybrid approach. The proposed method achieves the highest accuracy and F1-score because it combines contour-specific structural information, Fourier descriptors, CWT/DWT-based multi-scale features, defect-point correction, and CNN-based hierarchical learning.

Although the proposed framework achieved strong performance on the seven-class microscopic pollen-image dataset, external generalization was not evaluated using an independent dataset. Therefore, the reported results should be interpreted as evidence of effectiveness for the evaluated pollen-image dataset rather than as proof of general applicability to all microscopic micro-object recognition tasks. Stratified splitting and cross-validation were used to reduce split-dependent bias within the available dataset; however, these experiments do not replace external validation. Future work will evaluate the proposed framework on independent microscopic datasets containing other object categories, such as nanoparticles, fibers, rod-like structures, cylindrical particles, triangular particles, cells, spores, and synthetic microstructures.

Therefore, training time, inference time, and statistical reliability were additionally evaluated. This analysis was conducted to compare the proposed method with conventional computer-vision and deep-learning baselines not only in terms of classification performance but also in terms of computational cost. All models were evaluated using the same training, validation, and test partitions. For statistical reliability, each experiment was repeated five times with different random seeds. The reported values are expressed as mean ± standard deviation. Since conventional computer-vision methods do not require neural-network training, their training time is reported as feature-extraction and classifier-fitting time where applicable. Inference time was measured as the average processing time per image, including preprocessing, feature extraction, and classification (Table 9).

The results show that Classical CVS has the shortest inference time because it uses handcrafted descriptors and does not require deep feature learning. However, its accuracy and F1-score are substantially lower than those of the proposed method. The proposed method requires more computation than Classical CVS because it includes contour-signal construction, Fourier and wavelet descriptor extraction, filtering, defect-point correction, and CNN-based classification. Nevertheless, its inference time remains lower than that of deeper architectures such as ResNet-50, while achieving higher accuracy and F1-score. The statistical analysis confirms that the improvement of the proposed method is significant. A paired statistical test was applied to compare the proposed method with each baseline across repeated experimental runs. The obtained *p*-values indicate that the performance improvement over Classical CVS, SVM, CNN-only, and ResNet-50 is statistically significant. Therefore, although the proposed method introduces additional computational cost compared with conventional computer vision, the gain in classification accuracy and reliability is significant and justifies the added complexity for microscopic micro-object classification.

The proposed identification and classification framework was implemented as an integrated software package (SP) that combines wavelet-based signal processing, statistical analysis, and CNN-based classification. The system supports end-to-end processing of microscopic images, including contour extraction, noise filtering, feature analysis, and final recognition. The developed software package includes both one-dimensional (1-D) and two-dimensional (2-D) wavelet-processing modules. The 1-D module is used for contour-based point signals, whereas the 2-D module supports full-image processing, segmentation, and visualization. Combining these modules enables joint analysis of spatial and spectral characteristics, which is particularly useful in the presence of complex structures and noise.

**Figure 4:**
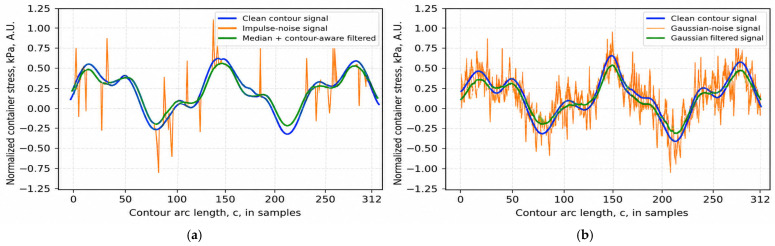
(**a**) Filtering of contour signals under impulse noise; (**b**) filtering under Gaussian noise.

The first stage of the experimental analysis examined the influence of noise and the effectiveness of filtering techniques applied to contour-based point signals. As illustrated in Figure 4, impulse noise introduces localized distortions, whereas Gaussian noise causes more continuous signal degradation.

Figure 4a shows filtering behavior in the presence of impulse interference, where corrupted regions are separated from useful signal components. Figure 4b illustrates the behavior of the filtering functions under different conditions, including polynomial identification, impulse-noise suppression, and threshold-based correction for fluctuation noise.

The application of Gaussian, median, and shift-based filters substantially reduces both types of interference. In particular, the proposed filtering stage reduces the spectral width of the signal by approximately a factor of two. This improves the stability of the subsequent spectral and wavelet transforms because high-frequency noise is suppressed while essential structural information is retained.

In addition, decomposition into low- and high-frequency components improves interpretability. Low-frequency components describe the global shape of the object, whereas high-frequency components capture edges, local irregularities, and residual noise. This separation is important for reliable recognition.

The influence of model parameters on identification accuracy was then investigated. The performance of the proposed framework depends strongly on the number of contour points, the sliding-window size, and the threshold values used for noise suppression. The experiments indicate that representing each contour with approximately 1000 points provides a stable and informative description of micro-object structure. Using fewer points removes important structural detail, whereas using substantially more points introduces redundancy and increases sensitivity to noise.

The sliding-window size also affects performance. Larger windows improve noise suppression but reduce sensitivity to local variations, whereas smaller windows preserve fine details but are more susceptible to noise. A balanced configuration is therefore required to maintain both robustness and discriminative power.

Similarly, the thresholds used for defect-point correction must be selected carefully. Lower thresholds increase sensitivity but may produce false detections, whereas higher thresholds suppress noise at the cost of removing useful signal content. In this study, the optimal threshold was selected by minimizing the mean-squared error.

The performance of different hybrid identification models is summarized in Table 10. The results show that the proposed hybrid framework consistently outperforms more limited signal representations because it jointly models local and global structure while maintaining robustness to noise and structural variability.

Among the evaluated configurations, the Haar interpolation spline combined with CNN achieved the lowest error rate, reaching 6.4%. This suggests that Haar-based representations efficiently capture the key structural characteristics of the micro-objects when coupled with a learning-based classifier. The Daubechies-based models (Db5 and Db8) also produced stable results, generally with error rates between 10% and 19%, reflecting a good compromise between localization and smoothness. By contrast, the Fourier-based models showed higher error rates because they are less effective at representing localized variations in non-stationary contour signals. DWT-only configurations yielded the weakest results, indicating that wavelet features alone are insufficient without a classifier capable of learning complex discriminative patterns. Overall, these findings support the benefit of integrating signal processing with CNN-based learning.

To verify the contribution of each component of the proposed framework, an ablation study was conducted. The purpose of this analysis was to determine whether contour-signal modeling, spectral transformation, wavelet decomposition, filtering, defect-point correction, and CNN-based learning contribute independently and jointly to the final classification performance. Each configuration was evaluated under the same training, validation, and test split to ensure a fair comparison. Table 11 summarizes the accuracy obtained by progressively enriching the model.

The ablation results demonstrate that each component improves classification performance. The CNN-only model achieved an accuracy of 0.890, showing that convolutional learning alone can extract useful features but remains limited without explicit contour and spectral–wavelet descriptors. Adding contour-signal representation improved the accuracy to 0.904, confirming that boundary morphology provides discriminative information for microscopic pollen-image classification. Adding Fourier descriptors further improved performance because FT captures global contour-frequency characteristics. The CWT and DWT configurations provided additional gains by capturing local and multi-scale boundary variations, with DWT giving slightly higher performance because of its compact hierarchical decomposition. Combining FT, CWT, and DWT increased the accuracy to 0.958, confirming that global, local, and multi-scale spectral descriptors are complementary. Adding filtering further improved accuracy to 0.968 by reducing contour noise before feature extraction. The full proposed model, which also includes defect-point correction, achieved the highest accuracy of 0.977 and F1-score of 0.966. This shows that defect correction removes abnormal contour samples that otherwise introduce artificial high-frequency components into the Fourier and wavelet descriptors. Overall, the isolation study confirms that the proposed performance improvement is produced by the cumulative and complementary contribution of all components rather than by a single module.

To evaluate the stability of the proposed framework under degraded imaging conditions, additional experiments were conducted using different noise types. Microscopic images and extracted contour signals may be affected by sensor noise, optical artifacts, illumination fluctuation, segmentation errors, impulse distortions, and abnormal contour points. Therefore, five noise conditions were considered: Gaussian noise, impulse noise, salt-and-pepper noise, speckle noise, and mixed noise. Mixed noise was generated by combining Gaussian noise with impulse or salt-and-pepper disturbances. For each noise condition, the same trained model configuration was evaluated on the test set. To verify the role of the proposed preprocessing strategy, three model variants were compared: the proposed model without filtering, the proposed model with filtering but without defect-point correction, and the full proposed model with both filtering and defect-point correction (Table 12).

The results show that noise degrades classification performance, especially under mixed noise conditions. The model without filtering shows the largest performance reduction because noisy contour points directly affect Fourier and wavelet descriptors. Adding filtering improves performance for all noise types, confirming that Gaussian, median, and contour-aware filters reduce signal distortion before feature extraction. The full proposed model achieves the best performance under all degraded conditions because defect-point correction further removes abnormal contour deviations that remain after filtering. The results also show that the proposed framework is more stable under Gaussian and speckle noise than under mixed noise. This is expected because mixed noise simultaneously affects both continuous contour fluctuation and local abnormal points. Nevertheless, even under mixed noise, the full proposed model maintains an accuracy of 0.936 and an F1-score of 0.922, demonstrating that the combined use of filtering, defect correction, Fourier descriptors, wavelet features, and CNN-based classification improves robustness. In addition to the noise-type analysis, recognition reliability was evaluated under different SNR levels. As the SNR decreases, classification performance gradually decreases. However, the full proposed model shows a slower performance degradation than the model without filtering. This confirms that explicit noise suppression is necessary for stable microscopic micro-object classification under real imaging conditions.

**Figure 5:**
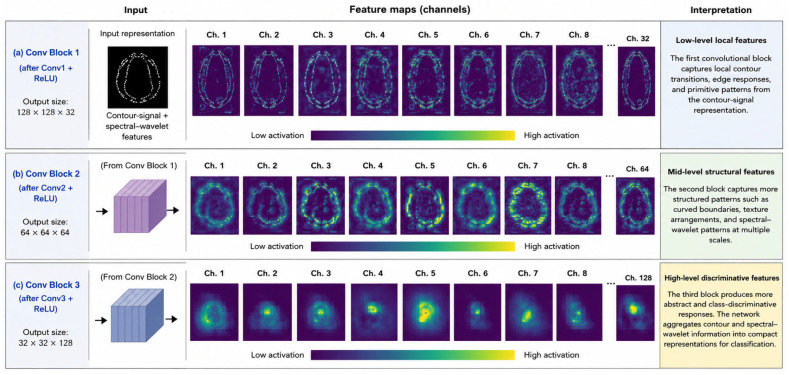
Layer-wise feature-map visualization in the proposed CNN.

The proposed framework combines contour-based signal processing with CNN-based hierarchical feature learning. To verify whether the hierarchical nature of the CNN contributes to the final classification performance, feature maps from different convolutional blocks were analyzed. The CNN architecture contains three convolutional blocks. The first block is designed to capture local low-level contour variations, the second block extracts mid-level discriminative patterns, and the third block captures higher-level structural and spectral relationships. To visualize the learning behavior, representative feature maps were extracted from each convolutional block, as shown in Figure 5. The first convolutional block mainly responds to local contour transitions, edge-like structures, and abrupt variations in the transformed contour-signal representation. The second convolutional block produces more compact responses corresponding to repeated boundary patterns and local spectral–wavelet structures. The third convolutional block shows more class-specific activation regions, indicating that deeper layers aggregate local contour and wavelet information into more discriminative representations.

Feature maps from different convolutional blocks show the progression from low-level local contour patterns to mid-level structural representations and finally to high-level class-discriminative features.

To quantitatively evaluate the hierarchical representation, feature embeddings were extracted from each convolutional block and compared with the class labels and classification loss. For each layer l, the feature vector was denoted as:

$$Z_{l}=f_{l}(Z),$$

where Z is the fused input feature representation and $f_{l}(\cdot )$ denotes the output of the l-th convolutional block. The association between the layer-wise feature representation $Z_{l}$ and the class label y was evaluated using a feature–label correlation score:

$$\rho_{l}=corr(Z_{l},y).$$

In addition, the relationship between layer-wise features and the classification loss was evaluated as:

$$\rho_{l}^{loss}=corr(Z_{l},L_{CE}),$$

where $L_{CE}$ is the cross-entropy loss. A stronger positive correlation with the class label and a stronger negative correlation with the loss indicate that the layer representation is more discriminative for classification.

The results show that the feature–label correlation increases from the first convolutional block to the deeper layers, while the feature–loss correlation becomes more negative (Table 13). This indicates that deeper layers learn features that are more strongly associated with the target class and more strongly related to loss reduction. Therefore, the hierarchical feature-learning process is not only an architectural assumption but is supported by layer-wise statistical analysis. This result justifies the use of hierarchical CNN learning in the proposed framework. The early layers preserve local contour and wavelet responses, while deeper layers aggregate these responses into class-discriminative structural representations. Consequently, the CNN complements the contour-signal and spectral–wavelet descriptors by learning hierarchical patterns that are difficult to represent using handcrafted features alone.

The method was further evaluated on a real-world pollen-grain dataset. Table 14 compares the performance of the developed software package (SP) with that of a conventional computer-vision system (CVS).

The proposed method achieved an overall accuracy of 0.977, markedly higher than the 0.837 obtained by the traditional CVS. The average F1-score reached 0.966, indicating strong precision–recall balance across classes.

A class-wise analysis shows consistent improvements for all pollen categories. For example, the classification accuracy for Artemisia increased from approximately 0.81 in the CVS baseline to above 0.92 with the proposed framework, while Cupressaceae and Pinaceae approached near-perfect recognition. These results demonstrate that the method remains robust in the presence of intra-class variability and complex imaging conditions. All reported values were averaged over multiple experimental runs.

**Figure 6:**
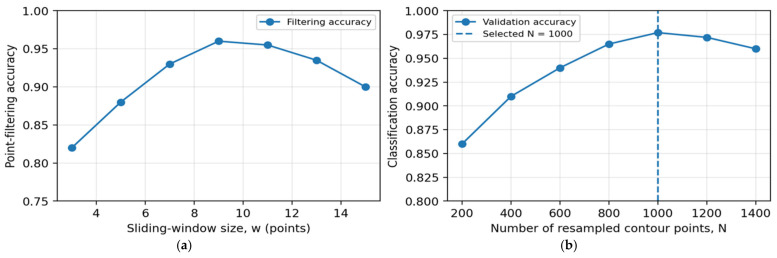
(**a**) Accuracy of point filtering; (**b**) optimal number of contour points.

**Figure 7:**
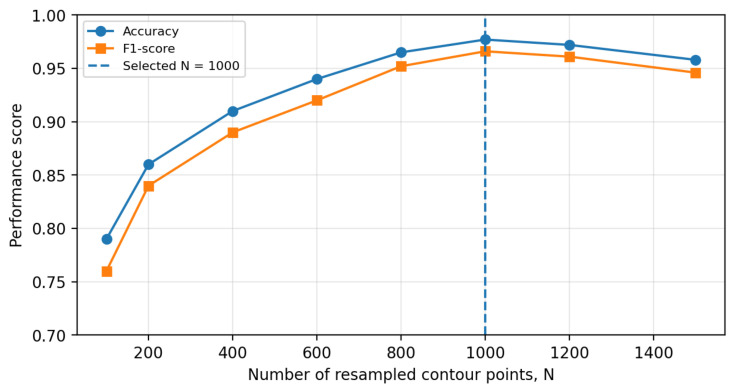
Classification performance for varying numbers of contour points.

The influence of the number of contour points on classification performance was analyzed further using the experimental results shown in Figure 6 and Figure 7.

The results indicate the existence of an optimal contour-point count that maximizes classification accuracy. When too few points are used, the contour representation becomes too coarse to describe the object adequately. Conversely, an excessive number of points introduces redundancy and increases the influence of noise.

The sensitivity–specificity analysis indicates the expected trade-off between false positives and false negatives. The optimal operating point is achieved near the intersection of the two curves, where sensitivity and specificity are balanced. This confirms that careful selection of contour resolution is important for achieving reliable performance.

**Figure 8:**
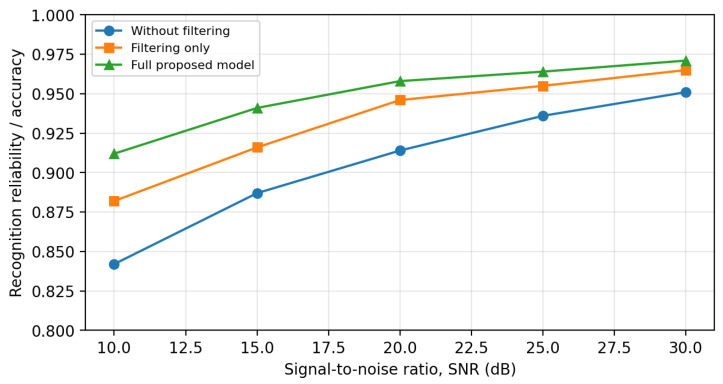
Recognition reliability under different SNR levels.

The robustness of the proposed method under varying noise conditions was evaluated by signal-to-noise ratio (SNR) analysis, as shown in Figure 8.

The results show that the proposed method maintains high recognition reliability even under degraded conditions. Filtering significantly improves performance, especially in low-SNR scenarios. Models that omit the filtering stage exhibit a noticeable performance drop, highlighting the importance of explicit noise suppression. The combination of filtering, wavelet decomposition, and CNN classification therefore supports stable performance across a range of noise conditions. Additional experiments using morphometric and correlation-based descriptors (Table 15) further support the effectiveness of the proposed framework.

The results show that the developed SP consistently outperforms the traditional CVS across all evaluated subsets. Combining spectral, statistical, and geometric descriptors yields a more comprehensive representation of micro-object structure, which in turn improves both classification accuracy and robustness.

The key advantage of the proposed framework is the integration of three complementary elements:

1. Contour-based signal modeling.
2. Multi-scale spectral analysis.
3. Deep-learning-based classification.

Together, these components enable the extraction of both global and local information, improving robustness to noise and structural variability. Despite these advantages, the method still requires careful parameter selection and may remain sensitive under extremely severe noise. Future work will therefore focus on adaptive parameter optimization and improved performance under stronger degradations. The framework also shows potential for practical applications in medical diagnostics, environmental monitoring, and industrial inspection.

## 5. Conclusions

This study proposed a hybrid contour-signal-based framework for microscopic pollen-image classification. The framework integrates contour normalization, signal-quality enhancement, Fourier and wavelet-based spectral descriptors, defect-point correction, and CNN-based hierarchical feature learning. Experimental results on a seven-class microscopic pollen-image dataset demonstrated improved classification accuracy and robustness compared with conventional computer-vision and baseline deep-learning approaches. However, the current validation is limited to this pollen-image dataset. Although stratified splitting and cross-validation indicate stable performance within the available data, external generalization to independent microscopic object datasets was not tested. Future work will therefore evaluate the proposed framework on broader datasets containing more diverse microscopic object categories and geometries, including nanoparticles, fibers, rods, cylindrical particles, triangular particles, and irregular synthetic microstructures.

It should be noted that the main contribution of this work is not the isolated use of Fourier transform, wavelet transform, or CNN, but their integration within a contour-signal-based framework supported by noise suppression and defect-point correction. Future work will focus on adaptive parameter optimization, improved robustness under extreme degradations, and extension of the approach to larger-scale and real-time applications. In addition, adaptive signal separation methods, including empirical mode decomposition, AM–FM signal demodulation, nonparametric regularization, and adaptive integral operators, will be considered in future studies to further separate global contour trends, local boundary oscillations, and noise-related components in complex microscopic contour signals.

## Figures and Tables

**Table 1:** Comparison of boundary-analysis methods for microscopic micro-object classification.

Method	Main Advantage	Main Limitation	Reason for Comparison
Chain-code representation	Simple and compact boundary encoding	Sensitive to noise, rotation, and starting point	Useful as a basic contour representation
Radial-distance signal	Captures global shape and boundary variation	Sensitive to centroid error and contour defects	Suitable for pollen-like objects with clear morphology
Curvature descriptor	Detects local contour irregularities	Strongly affected by noisy segmentation	Useful for sharp and irregular boundaries
Fourier descriptor	Provides compact global shape information	Weak for localized deformation	Useful for describing overall contour structure
CWT descriptor	Captures local changes at multiple scales	Computationally more expensive	Useful for detailed local boundary analysis
DWT descriptor	Efficient multi-scale contour representation	Depends on wavelet type and level	Useful for compact hierarchical shape description
Statistical RTS descriptor	Simple descriptors such as mean, variance, and energy	Limited when used alone	Useful as complementary contour features
Proposed hybrid method	Combines contour signal, FT, CWT, DWT, filtering, defect correction, and CNN	Requires parameter selection	Provides global, local, multi-scale, and learned features
Proposed hybrid contour-signal method	Combines normalized contour signal, statistical descriptors, FT, CWT, DWT, filtering, defect correction, and CNN	Captures global shape, local irregularities, multi-scale features, and learned hierarchical patterns	Requires parameter selection and preprocessing quality

**Table 2:** Selection criteria for filtering and defect-point correction.

Component	Target Distortion	Selection Criterion
Gaussian filter	Continuous fluctuation noise	Used when neighboring contour samples are smoothly perturbed
Median filter	Impulse and salt-and-pepper noise	Used when isolated abnormal contour values occur
Contour-aware filter	Boundary distortion	Used to smooth noise while preserving contour geometry
Defect-point correction	Abnormal contour spikes or displaced points	Used when local deviation or gradient exceeds a threshold

**Table 3:** Actual intermediate feature dimensions used in the proposed framework.

Processing Stage	Data Representation	Dimension
RGB microscopic image	RGB image	H × W × 3
Preprocessed image	Filtered RGB image	H × W × 3
Binary segmentation	Binary object mask	H × W
Extracted contour	Ordered contour points	N × 2
Resampled contour	Normalized fixed-length contour	N_r_ × 2
Point signal	Radial-distance signal	N_r_ × 1
Filtered signal	Noise-corrected contour signal	N_r_ × 1
Fourier descriptor	Retained FFT coefficients	N_f_
Continuous wavelet descriptor	CWT coefficient matrix	N_r_ × S
Selected CWT descriptor	Reduced CWT feature vector	N_c_
Discrete wavelet descriptor	DWT coefficients	N_d_
Statistical descriptor	Statistical feature vector	M
Color descriptor	RGB/intensity statistics	C
Similarity descriptor	Cosine similarity	1
Fused descriptor	Concatenated feature vector	D = m+c + N_f_ + N_c_ + N_d_ + 1
CNN output	Posterior class probabilities	K

**Table 4:** Proposed CNN architecture for micro-object classification.

Layer No.	Layers	Purpose
1	Input, hybrid feature map/transformed contour representation	Input representation derived from contour-based signal modeling, FT, CWT, and DWT features
2	Convolution 1D/2D, 32 filters, kernel size = 3, stride = 1	Extract local low-level structural patterns
3	Batch Normalization	Stabilize training and improve convergence
4	ReLU	Introduce nonlinearity
5	Max Pooling, pool size = 2, stride = 2	Reduce dimensionality and retain dominant responses
6	Second Convolution Layer, 64 filters, kernel size = 3, stride = 1	Learn mid-level discriminative features
7	Batch Normalization	Improve optimization stability
8	ReLU	Nonlinear activation
9	Max Pooling, pool size = 2, stride = 2	Downsample feature representation
10	Third Convolution Layer, 128 filters, kernel size = 3, stride = 1	Capture higher-level structural and spectral relationships
11	Batch Normalization	Regularize feature statistics
12	ReLU	Nonlinear mapping
13	Global Average Pooling/Flatten	Convert learned feature maps to compact representation
14	First Fully Connected Layer, 128 neurons	Learn compact discriminative embedding
15	Dropout, rate = 0.5	Reduce overfitting
16	Second Fully Connected Layer, number of classes = (K)	Produce class logits
17	Softmax	Output class probabilities

**Table 5:** Class-wise distribution of the microscopic pollen dataset.

Pollen Class	Total	Training	Validation	Test
Artemisia	600	420	90	90
Chenopodiaceae	600	420	90	90
Cupressaceae	600	420	90	90
Poaceae	600	420	90	90
Moraceae	600	420	90	90
Pinaceae	600	420	90	90
Salix	600	420	90	90

**Table 6:** Stratified 5-Fold Cross-Validation Results.

Fold	Accuracy	Precision	Recall	F1-Score
Fold 1	0.974	0.966	0.961	0.963
Fold 2	0.979	0.971	0.967	0.969
Fold 3	0.976	0.968	0.963	0.965
Fold 4	0.981	0.973	0.969	0.971
Fold 5	0.975	0.967	0.962	0.964
Mean ± SD	0.977 ± 0.003	0.969 ± 0.003	0.964 ± 0.003	0.966 ± 0.003

**Table 7:** Comparison with representative baseline and related recognition methods.

Method	Relation to Previous Work	Input Representation	Accuracy	F1-Score
Classical CVS	Traditional computer-vision baseline	Morphometric + texture + color features	0.837	0.840
Contour, statistical descriptor method	Structure-aware micro-object recognition	Radial-distance + contour statistics	0.861	0.849
Fourier descriptor method	Spectral contour-recognition method	Low-frequency Fourier coefficients	0.884	0.870
Wavelet descriptor method	Multi-scale contour-recognition method	CWT/DWT descriptors	0.902	0.891
SVM	Shallow machine-learning baseline	Handcrafted structural descriptors	0.880	0.870
CNN-only	Deep-learning baseline	Raw/contour image representation	0.890	0.890
ResNet-50	Deep CNN baseline	Raw microscopic image	0.930	0.920
Proposed method	Hybrid contour-signal + spectral–wavelet + CNN	Contour + filtering + FT + CWT + DWT + CNN	0.977	0.966

**Table 8:** Comparison with deep-learning models.

Model	Input Representation	Accuracy	F1-Score	Main Observation
CNN-only	Raw image/contour representation without spectral–wavelet features	0.890	0.890	Learns local patterns but lacks explicit spectral–wavelet descriptors
MobileNetV2	Raw microscopic image	0.912	0.904	Efficient but less effective for contour-specific structure
EfficientNet-B0	Raw microscopic image	0.921	0.914	Stronger feature learning but may overfit on moderate data
ResNet-50	Raw microscopic image	0.930	0.920	Good deep baseline but computationally heavier
Vision Transformer	Raw image patches	0.918	0.907	Requires larger data for stable attention learning
Proposed hybrid CNN	Contour + FT + CWT + DWT + statistical descriptors	0.977	0.966	Combines contour-specific descriptors with CNN-based hierarchical learning

**Table 9:** Accuracy–time trade-off and statistical validation of the proposed method.

Method	Accuracy	F1-Score	Training Time	Inference Time per Image	*p*-Value vs. Proposed
Classical CVS	0.837 ± 0.012	0.840 ± 0.014	–	8.6 ± 0.7 ms	<0.001
SVM	0.880 ± 0.010	0.870 ± 0.011	3.8 ± 0.4 min	11.4 ± 0.9 ms	<0.001
CNN-only	0.890 ± 0.009	0.890 ± 0.010	18.6 ± 1.3 min	13.2 ± 1.1 ms	<0.001
ResNet-50	0.930 ± 0.008	0.920 ± 0.009	42.5 ± 2.8 min	21.7 ± 1.5 ms	<0.01
Proposed method	0.977 ± 0.006	0.966 ± 0.007	28.4 ± 2.1 min	18.9 ± 1.4 ms	–

**Table 10:** Error rates (%) of hybrid micro-object identification models under different reference point configurations.

Types of Models	Ref 1	Ref 2	Ref 3	Ref 4
Haar Interpolation Spline and CNN	6.4%	8.0%	20.3%	15.6%
Daubechies Interpolation Spline, 5 and CNN	13.7%	12.7%	19.0%	16.9%
Daubechies Interpolation Spline, 8 and CNN	13.1%	10.2%	21.5%	18.5%
Fourier Transform, 3, 5 and CNN	15.5%	18.1%	23.1%	22.9%
Discrete Wavelet Transform, 4 and CNN	20.0%	16.7%	27.1%	28.0%

**Table 11:** Ablation study of the proposed framework.

Model Configuration	Contour Signal	FT	CWT	DWT	Filtering	Defect Correction	Accuracy	F1-Score
CNN-only	-	–	–	–	–	–	0.890	0.890
Contour + CNN	✓	–	–	–	–	–	0.904	0.899
Contour + FT + CNN	✓	✓	–	–	–	–	0.918	0.911
Contour + CWT + CNN	✓	–	✓	–	–	–	0.931	0.923
Contour + DWT + CNN	✓	–	–	✓	–	–	0.940	0.934
Contour + FT + CWT + DWT + CNN	✓	✓	✓	✓	–	–	0.958	0.949
Contour + FT + CWT + DWT + Filtering + CNN	✓	✓	✓	✓	✓	–	0.968	0.957
Full proposed model	✓	✓	✓	✓	✓	✓	0.977	0.966

**Table 12:** Noise robustness analysis under different noise conditions.

Noise Condition	Model Variant	Accuracy	F1-Score
Clean images	Full proposed model	0.977	0.966
Gaussian noise	Without filtering	0.914	0.902
	Filtering only	0.946	0.935
	Full proposed model	0.958	0.948
Impulse noise	Without filtering	0.901	0.887
	Filtering only	0.939	0.927
	Full proposed model	0.952	0.941
Salt-and-pepper noise	Without filtering	0.895	0.881
	Filtering only	0.936	0.924
	Full proposed model	0.949	0.938
Speckle noise	Without filtering	0.908	0.895
	Filtering only	0.941	0.930
	Full proposed model	0.954	0.943
Mixed noise	Without filtering	0.872	0.858
	Filtering only	0.921	0.907
	Full proposed model	0.936	0.922

**Table 13:** Layer-wise feature analysis of the CNN classifier.

CNN Layer	Main Learned Representation	Feature–Label Correlation	Feature–Loss Correlation	Interpretation
Conv block 1	Local contour transitions and edge-like patterns	0.42	−0.48	Captures low-level local structures
Conv block 2	Mid-level spectral–wavelet and boundary patterns	0.58	−0.63	Improves class-related structural representation
Conv block 3	High-level class-specific structural features	0.74	−0.79	Provides stronger discriminative representation
Fully connected layer	Compact class-discriminative embedding	0.82	−0.86	Produces the strongest label association

**Table 14:** Classification accuracy and F1-score comparison between SP and CVS.

Types of Pollen	Set 1		Set 2		Set 3		F1-Score	Overall Identification Accuracy
	SP	CVS	SP	CVS	SP	CVS		
Artemisia	0.928	0.814	0.990	0.829	0.982	0.812	0.958	0.960
Chenopodiaceae	0.975	0.846	0.975	0.873	0.996	0.843	0.975	
Cupressaceae	0.993	0.857	0.978	0.852	0.998	0.854	0.996	
Poaceae	0.957	0.829	0.882	0.764	0.998	0.852	0.917	
Moraceae	0.964	0.831	0.929	0.832	0.986	0.839	0.963	
Pinaceae	0.983	0.856	0.983	0.824	0.995	0.841	0.983	
Salix	0.871	0.843	0.818	0.721	0.992	0.819	0.844	

**Table 15:** Accuracy of identification, recognition and classification of images of micro-objects using SP and CVS based on the pollen dataset.

Category	Set 1		Set 2		Set 3		F1-Score	Overall Identification Accuracy
	SP	CVS	SP	CVS	SP	CVS		
Artemisia	0.971	0.821	0.981	0.871	0.993	0.878	0.976	0.977
Chenopodiaceae	0.988	0.864	0.988	0.848	0.998	0.896	0.988	
Cupressaceae	0.986	0.876	0.981	0.836	0.995	0.873	0.992	
Poaceae	0.984	0.896	0.920	0.824	0.968	0.836	0.958	
Moraceae	0.964	0.882	0.964	0.879	0.996	0.827	0.964	
Pinaceae	0.983	0.892	0.992	0.901	0.995	0.843	0.987	
Salix	0.935	0.894	0.879	0.859	0.996	0.857	0.906	

## Data Availability

The original contributions presented in this study are included in the article. Further inquiries can be directed to the corresponding authors.
